# A Review of Global Health Competencies for Postgraduate Public Health Education

**DOI:** 10.3389/fpubh.2017.00046

**Published:** 2017-03-20

**Authors:** Shailendra Sawleshwarkar, Joel Negin

**Affiliations:** ^1^Western Sydney Sexual Health Centre, Sydney Medical School Westmead, Marie Bashir Institute for Infectious Diseases, University of Sydney, Sydney, NSW, Australia; ^2^School of Public Health, Sydney Medical School, University of Sydney, Sydney, NSW, Australia

**Keywords:** global health, competencies, international health, postgraduate education, public health

## Abstract

During the last decade, the literature about global health has grown exponentially. Academic institutions are also exploring the scope of their public health educational programs to meet the demand for a global health professional. This has become more relevant in the context of the sustainable development goals. There have been attempts to describe global health competencies for specific professional groups. The focus of these competencies has been variable with a variety of different themes being described ranging from globalization and health care, analysis and program management, as well as equity and capacity strengthening. This review aims to describe global health competencies and attempts to distill common competency domains to assist in curriculum development and integration in postgraduate public health education programs. A literature search was conducted using relevant keywords with a focus on public health education. This resulted in identification of 13 articles that described global health competencies. All these articles were published between 2005 and 2015 with six from the USA, two each from Canada and Australia, and one each from UK, Europe, and Americas. A range of methods used to describe competency domains included literature review, interviews with experts and employers, surveys of staff and students, and description or review of an academic program. Eleven competency domains were distilled from the selected articles. These competency domains primarily referred to three main aspects, one that focuses on burden of disease and the determinants of health. A second set focuses on core public health skills including policy development, analysis, and program management. Another set of competency domains could be classified as “soft skills” and includes collaboration, partnering, communication, professionalism, capacity building, and political awareness. This review presents the landscape of defined global health competencies for postgraduate public health education. The discussion about use of “global health,” “international health,” and “global public health” will continue, and academic institutions need to explore ways to integrate these competencies in postgraduate public health programs. This is critical in the post-MDG era that we prepare global public health workforce for the challenges of improving health of the “global” population in the context of sustainable development goals.

## Introduction

In the post-MDG era, there is increasing recognition that health and sustainable development are inseparable in an increasingly globalized and interconnected world (1, 2). The complex interplay between humans, animals, and environment; dual burden of communicable and non-communicable diseases; emerging economies; and mobility of people are having profound political, social, and economic consequences (3). The concept of “global health” as the health of an interdependent global population is not only shaping our understanding of which and whose problems we tackle but also the way in which we educate students and design the global institutions that govern our collective efforts to protect and promote public health worldwide (3).

During the last decade, there has emerged a growing body of literature about global health. Based on MEDLINE searches, from 1996 to 2000, there were fewer than 50 publications with “global health” in either the title or abstract, which increased to more than 6,000 from 2011 to 2015. Global health has been defined in the context of various professional groupings such as medicine, nursing, oral health, pharmacy, allied health, and also medical subspecialties (4–10). Global health overlaps with international health and tropical medicine, and there are calls to explore similarities and differences between global health, international health, and public health (11, 12). The global aspect of this term has been debated, and several meanings have been discussed. Global may imply worldwide or transcending national boundaries, or it could signal the interdisciplinary nature of public health in a globalized world (2).

The generation of knowledge and its optimal use and application, especially in the field of global health and development, has garnered significant attention in recent years. There has been a dramatic growth in the number of educational programs that offer global health programs at undergraduate and postgraduate levels. A consensus definition for global health remains elusive. Commonly used is that of Koplan and colleagues who defined global health as “an area for study, research, and practice that places a priority on improving health and achieving equity in health for all people worldwide. Global health emphasizes transnational health issues, determinants, and solutions; involves many disciplines within and beyond the health sciences and promotes interdisciplinary collaboration; and is a synthesis of population-based prevention with individual-level clinical care” (12).

Educational competencies are informed by the needs of professional workforce and include a combination of knowledge, skills, and attitudes required for acceptable level of practice. Educational competencies are critical to curriculum development and evaluation, coordination across education programs, faculty development, and scholarship. The Institute of Medicine’s report “Who Will Keep the Public Healthy? Educating Public Health Professionals for the 21st Century” published in 2003 identified eight emerging areas significant to the future of public health education, which included global health, communication, cultural competence, and ethics (13). The report also recommended that competencies in these emerging content areas need to be identified and incorporated in graduate public health education (13). Calhoun and colleagues described the development of the Association of School of Public Health’s Core competency model for Master of Public Health (MPH) degree and discussed that the aim of these competencies was to provide knowledge, skills, and expected attributes of emerging public health professional (14). Recently, several reports including the Lancet Report on Health Professional education and the WHO report on “Transforming and Scaling up Health Professionals Education and Training” guidelines have highlighted the centrality of competency-driven education for bringing in educational reforms (15, 16). Calhoun and colleagues reviewed competency-based education program development in the context of global health workforce and discussed the status of competency model adoption across public health schools in the USA (17). This review analyzed the competency domains and outcomes using Blooms Taxonomy of educational outcomes showing that most competencies are in cognitive domain at a higher application level (17).

The recent literature in global health education has elicited several reviews that have sought to identify “core competencies” for various professional groups including medical, nursing, and public health education (5, 17–21). Academic institutions are also exploring the scope of their public health educational programs in the context of global health education to meet the demand for a global health professional in the context of the sustainable development goals. The American Association of Schools and Programs of Public Health Global Health Committee proposed a Global Health Competency Model, and the Consortium of Universities for Global Health (CUGH) global health subcommittee published four levels of interprofessional global health competencies (20, 21). There have been other attempts to describe global health competencies in the context of specific education programs and perspectives from stakeholders (22–25). The focus of these competencies has been variable with a range of different themes being described ranging from globalization and health care, analysis and program management, as well as equity and capacity strengthening. This review of the literature will provide a landscape of global health competencies and attempt to distill common competency domains to assist in curriculum development and integration in postgraduate public health education programs.

## Methods

### Search Strategy

A literature review was conducted in April 2016 using six electronic databases using the keywords: TITLE-ABS (Global Adj3 health OR international Adj3 health) AND (education* or training* or universit* or curriculum* or college*) AND (competenc* or skill* or outcome* or objective*). The search was limited to English language only, and all document types were searched with a focus on primary research studies, evaluation reports, conference abstracts, and all types of review articles.

Title/abstract was searched in the following databases: Ovid Medline (1946–present), Embase (1974–present), Global Health (1910–present), ERIC—Education (1966–present), EBM Reviews—Cochrane Database of Systematic Reviews (2005–present), and PsycINFO (1967–present). Additional search was undertaken using Scopus using keywords “global health” and “competenc*”to explore additional references.

Two reviewers (Shailendra Sawleshwarkar and Joel Negin) initially reviewed the titles and selected articles that discussed global health from an educational aspect for the abstract review. All shortlisted titles were directly imported into Endnote© version 7 (Thomson Reuters, Philadelphia, PA, USA). Duplicate results were then removed. During abstract review stage, studies related to specific medical, nursing, and medical subspecialties professions that were related to global health but not dealing with public health were excluded. After limiting abstracts to those related to global health from educational point of view, a full text review was conducted to identify those dealing with global public health education. At this stage, a detailed analysis was done of all those articles, and specific articles addressing global health competencies in the context of graduate public health education were identified. After going through the bibliographies of these articles a cross search was undertaken to identify any additional relevant articles.

Articles were discussed by two authors (Shailendra Sawleshwarkar and Joel Negin), and full review of the selected articles was undertaken. Information was extracted on global health competencies, year of publication, type of program—global health/international public health/public health/other, location of the review, method used for developing or describing competencies (if relevant), competency domains/themes, and list of competencies in each domain/theme.

Core competency articles were read in detail, and a list was generated with all competencies mentioned in these articles. A note was made if the article identified the competency domain directly in their article, and this was noted as “Yes,” and if this was not identified, then “No” was recorded. If the article described the competency domains in the context of learning outcomes but competency domain was not explicitly stated then this was recorded as “Implied.”

## Results

**Figure 1:**
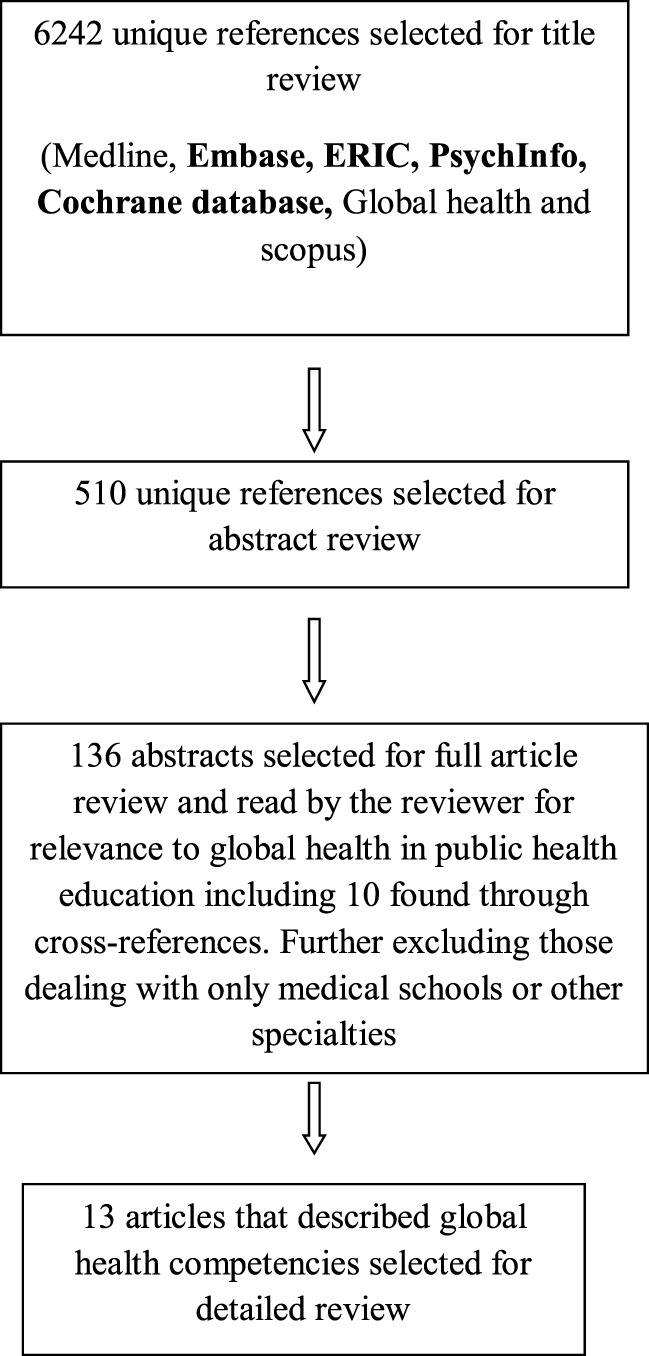
**Flowchart of included studies**.

Figure 1 presents a flow chart of the search strategy. The titles of 6,242 references were screened by 1 independent reviewer, and 510 unique references were selected for abstract review. After limiting abstracts to those that dealt with global health education from public health aspect and after excluding duplicates, this reduced the number of abstracts to 126. Additional search with and cross references yielded 10 additional abstracts that were added and resulted in a total of 136 for a review. Fifteen articles described competencies either as a focus of the article or in the context of a program. Two of these were not included in the final set as one of them described the same program and as another article focused on global health research competencies. Thirteen articles described competencies in the context of public health education and are summarized in the Table 1.

**Table 1:** **Summary of 13 included articles in global health competency review**.

Study working title/country	Description of competency domains/themes	Type of study	Comment/s
1. Global health education in UK universities (19)	Global burden of disease; Epidemiology of tropical diseases; Population mobility; Social studies (broadly defined to include culture, social responsibility, humanism, and social change); Socioeconomic determinants of health; Health-care services; Health systems; Global governance; Environmental change; Population health; Globalization; Human rights and ethics; International development; Monitoring and evaluation; Management and leadership; Policy analysis and development	Review of literature among UK universities offering global health programs	Five out of six articles reviewed focused on global health for medical education

2. Improving global health education—USA (20)	Capacity strengthening Collaborating and partnering Ethical reasoning and professional practice Health equity and social justice Program management Sociocultural and political awareness Strategic analysis	Global health competency model developed through a multistage modified-Delphi process by the Association of Schools of Public Health	Focused on broader global health competencies for public health students

3. Being global in public health practice and research—Canada (18)	*Category: Public Health Sciences*Demonstrate knowledge of: Historical and present north–south power dynamics; social and political contexts; and determinants of health. Linkages between local and global health problems. International organizations, their interactions, and their effects on local actions for health. Category: Policy and Program Planning, Implementation, and Evaluation Work effectively and responsibly in low-resource settings to promote sustainable interventions for global health Category: Partnerships, Collaboration, and Advocacy Foster self-determination, empowerment, and community participation in GH contexts. Actively recognize the interaction between political and economic history, power, participation, and engagement globally. Contribute to improving health equity at multiple levels, through systems changes. Category: Diversity and Inclusiveness Critically self-reflect upon one’s own social location and appropriately respond to others in their diverse locations. Communicate effectively across disciplines and cultures. Demonstrate commitment to global equity, social justice, and sustainable development. Category: Communication Create social spaces for dialog between stakeholders across jurisdictions. Category: Leadership Demonstrate willingness to be mentored across borders. Mentor others and develop long-term relationships of trust locally and globally. Educate oneself about global health issues on an ongoing basis	Commentary on global health practice and research competencies	Includes competencies for public health practice and also research

4. Identifying interprofessional global health competencies—USA (21)	Global burden of disease. Globalization of health and health care. Social and environmental determinants of health. Capacity strengthening. Collaboration, partnering, and communication. Ethics Professional practice Health equity and social justice. Program management. Sociocultural and political awareness. Strategic analysis	Consortium of Universities for Global Health subcommittee through literature review, discussions, voting, and consensus	Describes interprofessional global health competencies at various levels including basic and program level (listed here)

5. Developing competencies for graduate school curriculum in international health—USA (22)	Identify, analyze, and challenge power structures. Describe the major underlying and proximate determinants of adverse health in developing countries. Apply community development skills, policy advocacy, and communication strategies to promote public health, while using human rights concepts and instruments to promote social justice. Describe the burden of the most important health problems. Be able to assess the appropriateness of intervention strategies. Evaluate and establish priorities to improve the health status of populations in low-resource settings. Incorporate qualitative, quantitative, and operations research skills to design and apply reliable, valid, and ethically sound research. Use collaborative and culturally relevant leadership skills. Analyze and explain the role of transnational networks and global institutions. Design, manage, and evaluate programs in developing countries in close collaboration with local institutions to assure equitable access to quality health care. Design practical, culturally relevant, and communication programs. Analyze and explain the economic, social, political, and academic conditions that can produce a strong health workforce	University of Washington (UW) international health program competencies through literature search, looking at other programs, expert reviews, and faculty and student survey	Describes competencies in the context of an international health educational program

6. Competency-based curricula to transform global health—USA (30)	*Knowledge*: Upstream socioeconomic and environmental determinants of health. Systems thinking—health care and political systems, capacity building *Skills*: Analytic skills—epidemiology, monitoring, and evaluation, Management and leadership skills—financial management, collaboration and teamwork, ability to work in different cultures Policy analysis and development skills—“political savvy”	26 in-depth interviews with global health leaders	Article also explored training approaches and recruitment priorities. Discussion on interdisciplinary training, interprofessional collaboration, and implementation science

7. A case-based problem-based learning approach to prepare master of public health candidates for complexities of global health—USA (24)	*Background in global health*: Describe historical, economic, political, social, and cultural factors that influence the health of populations around the world. *Critical thinking*: Critique and design global health approaches affecting the health status of individuals, communities, and populations around the world. *Public health ethics*: Evaluate and apply public health ethical frameworks to design programs, policies, and interventions intended to improve health services and health status of individuals, communities, and populations. *Systems thinking*: Assess and incorporate spheres of influence or systems that affect global health challenges into policies to improve the health status of individuals, communities, and populations	Overarching Competencies for Core Global Health Course GH501: “Global Challenges and Opportunities,” at Hubert Department of Global Health at the Rollins School of Public Health—Emory University, Atlanta, GA, USA Review of competencies and staff, employers and student inputs in context of curriculum review	Describes pedagogical approach, course structure, and logistics for implementing global health course

8. Identifying competencies for Australian health professionals working in international health—Australia (23)	*Basic public health skills*: Specific skills in public health, disease control and prevention, health promotion skills *Management skills*: Management Policy, planning, and development skills Program planning, design, implementation, M&E Multidisciplinary teamwork/team building Communication skills—negotiation, mentoring, conflict resolution, advocacy, and liaison *Communication*: English proficiency and local or second language Written skills Consultation or advisory skills Cross-cultural skills Generic cross-cultural skills Collaboration and partnership skills *Analytical and Research skills*: Analytical skills Research skills including research ethics	Literature review, job competencies review, key stakeholder interviews	Focus is on Australian professionals working in low- and middle-income countries (LMIC)

9. Defining and developing a global public health course for public health graduates—Nepal and Australia (29)	*Emerging areas relevant to global public health knowledge and skills*: Globalization and health Disease burden Ethics and vulnerable groups Culture, society, and politics Policy and management *Relevant units to include in any global public health specialty course*: Primary health care and health promotion in LMIC Comparative health system Program/project development, management, and evaluation Management, leadership, teamwork Globalization and health Maternal and child health Global disease burden Culture, social system, social development, and health	Literature review and discussion about Relevant units to include in any global public health specialty course and rationale	Described mainly in the context of additional specialist competencies relevant to the context of LMIC that are needed to work in this field

10. Graduate Global Public Health Education—Canada (28)	Understand the political economy of global health issues. Bring a determinants-of-health and population health perspective to problem analysis, policy development, and project design. Be cognizant of the linkages between local and global health problems. Work within the mandates, roles, and approaches of international organizations. Build coalitions and work in partnership with the NGO sector and local community organizations. Be sensitive to cultural differences and adapt methods to local contexts. Understand broad ethical issues as they relate to equity globally. Apply appropriate ethical approaches to international, country level, and local projects	The competencies are described as activities and outcomes in relation to student’s prior experience at the “Global Health Concentration Model” at University of Toronto	Competencies are described in the context of review of a concentration model of global health education along with description of student backgrounds and outcomes

11. Fifteen years of the tropEd Masters in international health programme—Europe with international partners (25)	Analyze factors that influence health Monitor and evaluate interventions Collaborate across disciplines and borders Identify research needs, analyze results Formulate responses to complex international issues Identify the influence of globalization on population health	Competencies described in the context of alumni survey of the relevance of the competencies gained by the Masters in International Health and perceived strengths and weaknesses of the program	Review of a program with European and other international partners from LMIC

12. The Pan American Health Organization (PAHO) and international health—Americas (26)	The six main competencies stressed by the Program are Situational analysis: the ability to analyze a situation in-depth so as to intervene successfully. Policy formulation and decision-making: the capacity to develop and influence policies and strategies conducive to life and human health. Negotiation and advocacy: the ability to understand and direct change processes in relation to a given problem or challenge that is shared by different groups or institutions. Project management and cooperation: the ability to develop and establish relationships and reach collaborative agreements that are mutually beneficial in order to achieve specific objectives. Production and dissemination of information: the ability to develop and communicate innovative information about international health. Communication: the ability to formulate an argument and communicate it effectively to key stakeholders in order to achieve a desired outcome	Competencies described in the context of Leaders in International Health Program “Edmundo Granda Ugalde.” Training Program in International Health developed by PAHO	Review of a program in Americas

13. Toward defining interprofessional competencies for global health education—USA (27)	To identify contextually relevant qualitative and quantitative information from the sciences, social sciences, and the humanities to inform global health work. To read and interpret relevant literature from the sciences, social sciences, and humanities. To practice ongoing discernment in relation to one’s own interests, strengths, and values. To appreciate natural, cultural, and human diversity. To be able to take the perspective of others (both other professionals and persons from other cultures or contexts). To be able to compare and contrast systems of care and the social production of health and well-being in different settings. To translate research into practice. To practice leadership and effective teamwork. To effectively communicate ideas about health and well-being to other professions, community leaders, and the general public. To optimize the potential of one’s scope of practice within the context of a team. To be able to articulate shared goals, ethics, and values within diverse teams. To demonstrate established habits for self-guided, ongoing learning in relation to global health policies, focus regions, or countries, and topical areas of interest	These competencies were prepared by the author in preparation for a roundtable on interprofessional global health competencies at the University of Maryland Baltimore 2013 Based on the UW–GHI experience the Association of Schools and Programs of Public Health Global Health Competency Model, Interprofessional Education Collaborative Core Competencies for Interprofessional Collaborative Practice (IPEC Competencies), and the Framework for Twenty-First Century Civic Learning and Democratic Engagement published by the National Task Force on Civic Learning and Democratic Engagement	Focus on the interprofessional skills competency domain for graduate global health education

### Article Characteristics

All the 13 shortlisted articles that described global public health competencies were published between 2005 and 2015 with 6 from the USA, 2 each from Canada and Australia, and 1 each from UK, Europe, and Americas (18–30). A range of methods were utilized to describe competencies and included literature review, interviews with experts and employers, surveys of staff and students, and description or review of an academic program.

Jogerst and colleagues described the work of Global Health Competency Subcommittee of CUGH to identify interprofessional global health competencies and proposed 2 competency levels with 13 competencies across 8 domains for the Global Citizen Level and 39 competencies across 11 domains for the Basic Operational Program-Oriented Level (21). Ablah and colleagues described a Global Health Competency Model developed through a multistage modified-Delphi process and described 7 domains and 36 competencies that complement the foundational public health competencies (20). Pfeiffer and colleagues conducted 26 semi-structured interviews with global health practitioners and leaders about the competencies and curriculum for global health professionals of the future and identified key knowledge (health systems and determinants of health) and skills (analytical, leadership and management, and policy development) areas (30).

A few articles described global health competencies in the context of either a master’s program in international health or tropical medicine or global health component of a program and included those at the course review at the Emory University (24), alumni survey at the University of Toronto (28), program review of international health program at the Pan American Health Organization (26), alumni survey of program run by the tropEd network (25), and curriculum development through review and survey at the University of Washington (22).

### Global Health Competency Domains

**Figure 2:**
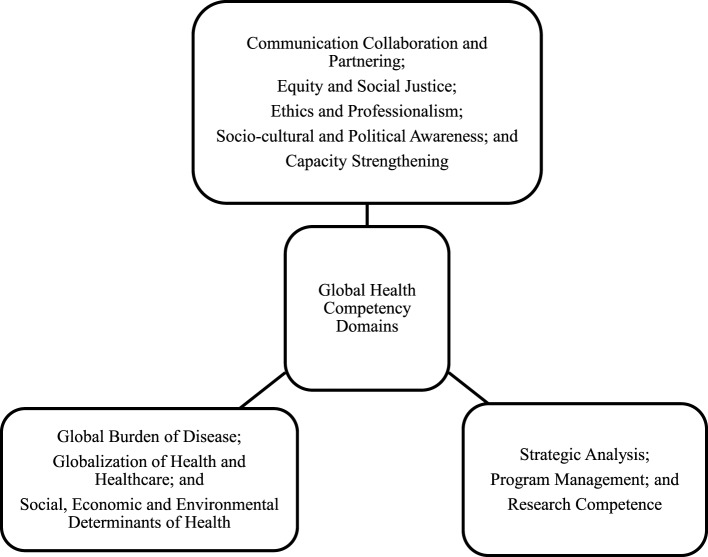
**Global health competency domains for public health education**.

The 13 articles listed various competency domains and described them—these are summarized in Table 1. These competency domains primarily refer to three main aspects, one that focuses on knowledge aspects of health and disease with inclusion of burden of disease and the determinants of health. A second set focuses on core public health skills including policy development, analysis, and program management. Another set of competency domains could be classified as “soft skills” with a combination of knowledge, skill, and attitude and include collaboration, partnering, communication, professionalism, capacity building, and political awareness. These 3 aspects of the 11 competency domain grouped as predominantly knowledge based, skill based, or a combination of knowledge, skill, and attitude are depicted in Figure 2. The list of competencies was reviewed, and similarly defined competencies were included under the same domain. There were some differences between articles regarding how these competencies were described and how they were grouped. This was more pronounced in the competency domains that discussed soft skills. Competency domains that were described by at least 6 of the 13 articles selected for review were included in the final list of 11 core competency domains along with a summary of the key elements of the identified competency domains distilled from selected articles (Table 2). Table 2 also lists the category (knowledge, skill, or attitude) of each competency domain as per the Bloom’s Taxonomy for educational objectives (31).

**Table 2:** **Core competency domains or themes for global health education and summary of the key elements of the identified competency domains distilled from selected articles**.

Competency domain	Knowledge (K), skill (S), and attitude (A)	Key elements of the competency domain
Domain 1: Global Burden of Disease	K	Basic understanding of burden of disease in all setting—high, middle, and low income including magnitude, distribution, and variations. Ability to use available data to validate the health status of the population

Domain 2: Globalization of Health and Health Care	K	Understanding of different health systems along with understanding of global health-care trends, human resources for health, and role of multiple stakeholders in planning and delivery health services. Understanding influence of globalization on health and be cognizant of linkages between local and global health

Domain 3: Social, Economic, and Environmental Determinants of Health	K	Understand social, economic, and environmental factors as determinants of population health. Key determinants of health and their impact on access to and quality of health services in different contexts and apply it to policy development and problem analysis

Domain 4: Capacity Strengthening	K, S, and A	Sharing of knowledge, skills, and resources to enhance public health programs to build human resource capacity and improve infrastructure. Strengthen community capabilities, build community partnerships, and with community integration improve health of individuals and communities. Analyze the economic, social, political, and academic conditions and address barriers to produce a strong health workforce

Domain 5: Ethics and Professionalism	K, S, and A	Understanding of and an ability to resolve common ethical issues and challenges that arise when working within diverse economic, political, and cultural settings to address global health issues. Evaluation and application of internationals standards and public health ethical frameworks in these settings. Demonstrate integrity, regard, and respect for others in all aspects of professional practice and optimize the potential of one’s scope of practice within the context of a team

Domain 6: Communication, Collaboration, and Partnering	S and A	Effectively communicate ideas about health and well-being to other professions, community leaders, and the general public. Communication skills including negotiation, mentoring, conflict resolution, advocacy, and liaison. Multidisciplinary teamwork and team building and working in close collaboration with local institutions to design, manage, and evaluate programs in developing countries

Domain 7: Health Equity and Social Justice	K and S	Apply social justice and human rights principles in addressing global health problems. Demonstrate commitment to global equity, social justice, and sustainable development

Domain 8: Program Management	K and S	Design, implement, and evaluate global health program to improve health of individuals and populations in a sustainable manner. Apply project management techniques throughout program planning, implementation, and evaluation. Ability to develop and establish relationships and reach collaborative agreements that are mutually beneficial in order to achieve program objectives

Domain 9: Sociocultural and Political Awareness	S and A	Ability to work effectively within diverse cultural settings and across local, regional, national, and international political landscapes. Being “Political savvy”—understand historical and present north–south power dynamics and social and political contexts

Domain 10: Strategic Analysis	S	To conduct situational analysis and bring systems thinking and determinants-of-health and population health perspective to analyze a diverse range of complex and interrelated factors to develop context-specific intervention to improve global health issues

Domain 11: Research Competence	S and A	Core public health research skills to incorporate qualitative, quantitative, and operations research skills to design and apply reliable, valid, and ethically sound research to identify innovative solutions for global health problems. Additional specific global health research competencies include identification of actionable determinants, involving communities, partnering with local institutions, and respecting cultural diversity. Translating research to policy and programs

**Figure 3:**
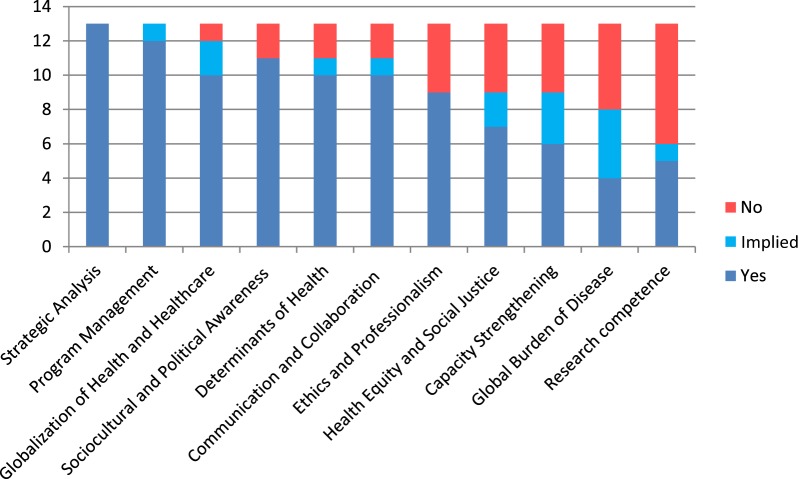
**Comparison of frequency of core competency domains/themes in selected articles (*n* = 13)**. Yes—directly describes competency domain. Implied—implies the identified competency domain. No—does not describe competency domain.

Analysis of the frequency of competency domains identified by the selected articles is shown in Figure 3 with domains 2, 8, and 10 being the most commonly identified domains. The competency domains that were mainly categorized as knowledge according to Bloom’s Taxonomy included Global burden of Disease, Globalization of Health and Health care, and Social, Economic, and Environmental Determinants of Health. Those that could be categorized as both skill and attitude included mainly “soft skills” such as communication, collaboration and partnering, ethics and professionalism, capacity strengthening, and sociocultural and political awareness.

Although this list captures the most of the competencies described in the selected articles, there were few competencies that were not included as separate domains in the 11 core competencies. These included those with specific focus on disease conditions or groups such as maternal and child health or travel medicine or some components of health systems or broader themes such as primary health care, health promotion, or global governance.

## Discussion

Health is central to the discussions about achieving sustainable development, and there are calls to review the role of public health in this era of globalization through the recently proposed Global Charter for the Public’s Health by the World Federation of Public Health Associations (32). Jenkins and colleagues argued for new conceptualization, review, and evaluation of “global public health” and also argued for curriculum change in public health education for the global public health workforce (33). Global health education has attracted increasing interest from academic institutions in the last decade. Current discussion about global health also presents an opportunity to explore integration of globalization, equity, political awareness, and interconnectedness in the public health curriculum in both high-income and low-income settings.

In this review, we identified 11 core competencies that may be relevant to postgraduate public health education. These competencies comprised those mainly dealing with knowledge such as global aspects of burden of disease and health care and mainly skill-based domains such as core public health skills of strategic analysis, and program management. The competencies also included those dealing with attitude and skill with key underlying themes of equity, ethics, professionalism, and human rights along with a set of “soft skills” such as communication, collaboration, partnering, and capacity strengthening and leadership were also identified. The competencies belonged to lower cognitive domains of ‘understand’ and ‘apply’ and also included a number of competencies with higher order cognitive domains of ‘analysis’ and ‘evaluation.’ The articles described mainly the knowledge aspects of health and disease and core public health skills of management and analysis [Harmer et al., Hagopian et al., and Karkee et al. generally did not include soft skills (19, 22, 29)]. Those articles that put more emphasis on “soft skills” typically did not include global burden of disease, but this may be due to assumption that it is a part of core public health knowledge (24, 26). The interprofessional competencies described by Jogerst et al. described two levels of competencies—global citizen level and basic program-oriented competencies (21). Global citizen level competencies include those that focus on health and disease along with communication skills and sociopolitical and cultural awareness at the global citizen level. At the program level these include program management, capacity strengthening, and strategic analysis, which include core public health competencies but also add a set of skills that also described by Ablah et al. in the global health competency model (20, 21). The Global Health Competency Model described by Ablah et al. specifically mentioned that the global health competency domains should be constructed upon the foundational MPH core competencies (20).

Global health competencies have been described for many professional groups—medical (4, 7, 34), nursing (5), and allied health (6). There seem to be some differences in the core competency domains for clinical health professionals and public health professionals: those for medical professionals focus more on the global burden of disease and health-care services. In a large-scale consultation on global health competencies for UK postgraduate doctors in the United Kingdom, involving over 250 diverse stakeholders identified 5 key competencies, which focused on global epidemiology, determinants of health and health systems along with global governance and ethics/human rights (4). There is significant overlap in the competency domains with the MPH core competencies (version 2.3) by the United States Association of Schools of Public Health in 2006 (14), but additional “soft skills” can be seen as building on the core public health competencies (20). The new soft skills such as political awareness, communication, and collaboration are an important tool in today’s globalized and politicized field of health.

The development of competencies allows for deeper thought on the definition and practice of global health. The similarities and differences in competencies are a good opportunity for academic public health institutions to engage in reviewing curriculum regarding knowledge, skill, and attitude required to prepare the global health professional. In their effort to define the field, Wernli and colleagues used a term “academic global health (AGH)” and suggested that it integrates the three traditional areas of health care, international health, and public health (35). They defined AGH as “within the normative framework of human rights, global health is a system–based, ecological and transdisciplinary approach to research, education, and practice which seeks to provide innovative, integrated, and sustainable solutions to address complex health problems across national boundaries and improve health for all” (35). The key dimensions described in this paper broadly deal with set of competency domains we have described in our paper including core knowledge areas in health care, core skills in public health, and set of “soft skills”. These “new public health professional skills” have been highlighted in a recent paper describing the collaborative consultation by the World Federation of Public Health Association about how to adapt public health to a future role in global health (36). Global health education in public health programs needs to explore ways to incorporate the soft skills in the curriculum in addition to the technical focus on knowledge and skills to prepare the public health professional to face challenges of global health in the context of sustainable development.

Our review has a few limitations. There are a relatively few articles published about global health competencies, and hence we have also included articles that described competencies in the context of postgraduate programs in international health. We also recognize that global health competencies in individual programs in school of public health may not be published but are important, and we are planning to review this information in future. This review only focused on literature published in English and so may have overlooked information that was published in other languages. In this review we mainly focused on domains of global health competencies and not detailed sub competencies and learning outcomes for each domain and learning outcomes and more detailed analysis of the identified competency domains. We plan to conduct the detailed analysis in the context of postgraduate public health educational program in the Asian region.

Most of our articles discussed competencies based on programs or discussions in developed countries with some limited representation from the Asia. While the concept of global health originated in the developed world, due to the nature of global health, the long-term relevance and success of education in global public health education depends on the adaptation of the curriculum in both high-income countries and low- and middle-income countries (LMIC) (35, 37). In a recent review, Rabbani and colleagues have argued that schools of public health in LMIC can contribute to overcoming several public health challenges faced by LMIC by building the capacity of cadres of competent and well-motivated public health workforce including educators, practitioners, and researchers (38).

## Conclusion

This review presents the landscape of defined global health competency domains for postgraduate public health education. The discussion about use of “global health,” “international health,” and “global public health” will continue, and academic institutions need to explore ways to integrate these competencies in postgraduate public health programs. This is critical in the post-MDG era that we prepare global public health workforce for the challenges of improving health of the “global” population in the context of sustainable development goals.

## Author Contributions

SS and JN were involved in conception and design of the review; involved in the interpretation of the data; approved the final submitted version. SS worked on the first draft with review by JN.

## Conflict of Interest Statement

The authors declare that the research was conducted in the absence of any commercial or financial relationships that could be construed as a potential conflict of interest.
